# Gout of the temporomandibular joint and review of the literature

**DOI:** 10.1259/bjrcr.20220046

**Published:** 2022-10-05

**Authors:** Joel Hng, Sankar Manchella, Ernest Lekgabe

**Affiliations:** 1 Department of Oral & Maxillofacial Surgery, The Royal Melbourne Hospital, Parkville, VIC, Australia; 2 Department of Radiology, The Royal Melbourne Hospital, Parkville, VIC, Australia

## Abstract

Gout is a disease characterised by abnormal deposition of monosodium urate crystals, typically affecting the extremities. This report describes a rare case of gout affecting the left temporomandibular joint with erosion of the skull base. A diagnosis of gout was suspected based on CT and MRI and confirmed with CT-guided biopsy. The temporomandibular joint is an uncommon location for a first presentation of gout, with very few cases documented and only three cases of skull base involvement reported in the English literature previously. Given its radiological appearance, it can easily be misdiagnosed as other erosive arthropathies or malignancy. Our paper highlights an unusual location for the first and only manifestation of gout and offers some diagnostic and treatment ideas that may help clinicians to identify and manage this disease.

## Summary

Gout is a disease characterised by the saturation of monosodium urate (MSU) crystals in the extracellular fluid, most commonly affecting joints of the lower limb, and rarely the temporomandibular joint (TMJ).^1^ There are only three cases in the English literature where erosion into the skull base is reported and in two of these cases, the patients underwent surgical treatment.^2–4^ The pathophysiology of gout results from hyperuricaemia, either as a result of overproduction, underexcretion or a combination of both.^5^ Another closely related condition is pseudogout, which is caused by calcium pyrophosphate dihydrate deposition.^5^ Pseudogout of the TMJ has been reported more often in the literature, with seven cases involving erosion into the skull base.^6^ Gout of the TMJ presents clinically in a similar way to pseudogout, but the difference is the aetiology and long-term treatment. The treatment for gout of the TMJ involves urate lowering medication as first-line management, and surgery if the patient continues to be symptomatic or has reduced jaw function.^7^

Due to the rare nature and the limited literature on gout of the TMJ, clinicians may not consider this diagnosis, resulting in suboptimal or delayed treatment. This case report shows the importance of considering this condition, especially if the skull base is involved to provide appropriate medical and surgical treatment. It presents new information and points of view on a known rare disease, which include diagnosis and treatment of gout of the TMJ.

## Clinical presentation

A 77-year-old female was referred to our unit with a 3 year history of constant and progressive left TMJ pain. Her comorbidities include ischaemic heart disease, thyroidectomy for multinodular goitre, asthma, chronic kidney disease, irritable bowel syndrome, atrial fibrillation and anxiety but no history of any inflammatory arthropathies. Her medications include amiodarone, metoprolol, mebeverine, frusemide, thyroxine, venlafaxine, sodium valproate, quetiapine and theophylline. Clinical examination revealed a notable swelling over the left TMJ, that was tender to palpate. She had no neurological deficits and no limitation in mouth opening. There was no palpable cervical lymphadenopathy. She was fully edentulous.

## Investigations

Routine blood examination revealed a CRP of 77.2 mg l^−1^ (normal range <3.0) and low eGFR of 21 mL/min/1.73 m^2^ in keeping with her chronic renal impairment. The remainder of the blood examination was unremarkable. A serum urate was not performed as gout was not suspected at that stage.

**Figure 1.**
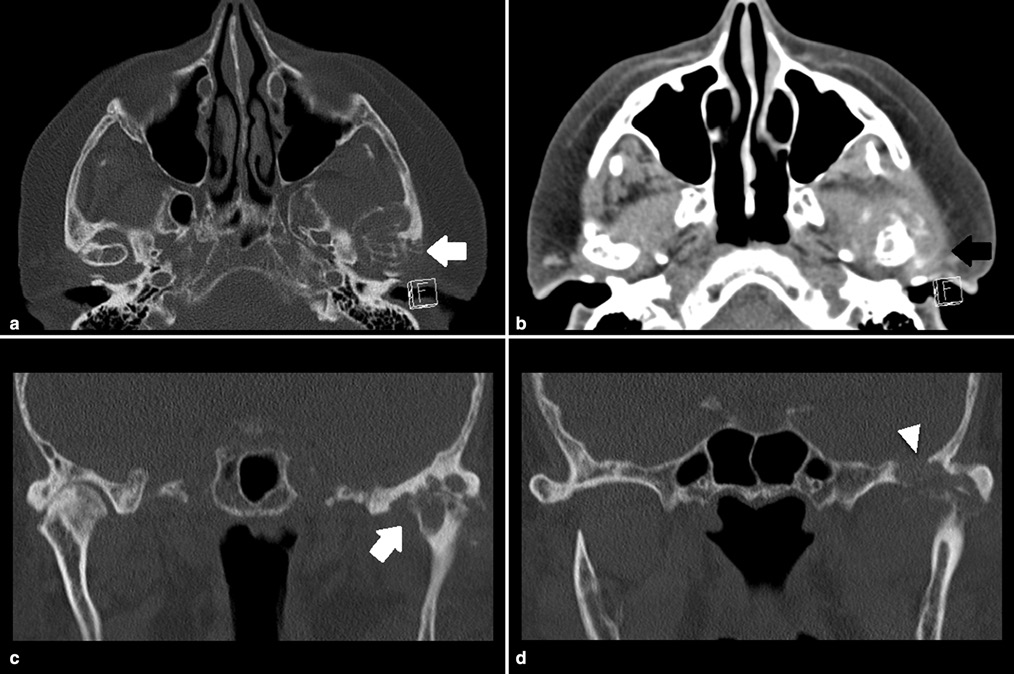
CT images showing relatively well-defined left TMJ articular and juxtaarticular erosions (white arrows) with through and through defect of the skull base (arrow head) and surrounding dense soft tissue thickening (black arrow). TMJ, temporomandibular joint.

**Figure 2.**
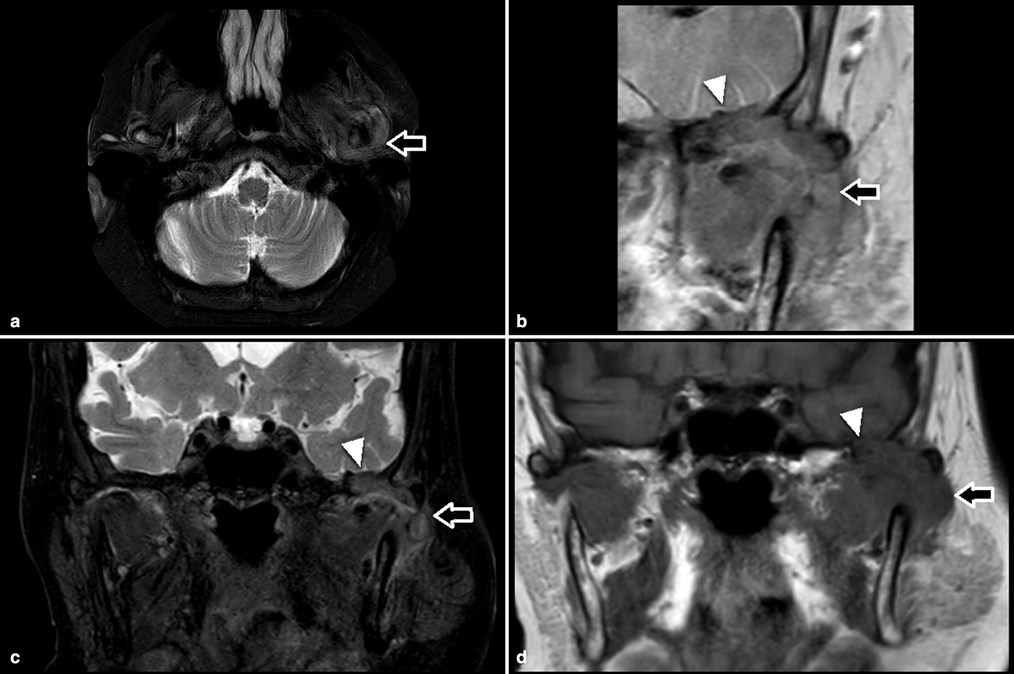
Axial and Coronal fat suppressed T2 (**a and c**), targeted coronal PD (**b**) and coronal T1 (**d**) MRI images showing erosions with through and through defect of the skull base (arrow head) and surrounding lobulated soft tissue thickening (arrows). PD, proton density.

CT and MRI were performed without contrast (due to the patient’s poor renal function) and revealed a destructive arthropathy of the left TMJ with relatively well-defined articular and juxtaarticular erosions associated with surrounding dense lobulated soft tissue thickening. The erosions caused a through and through defect of the skull base (Figures 1 and 2). The adjacent brain parenchyma was normal. Initially, the oral and maxillofacial surgery team considered malignancy due to the degree of joint destruction and skull base erosion. However, after consulting with a head and neck radiologist, a joint arthropathy was suspected because the imaging findings were centred in and around the joint and involving either side-of the joint relatively equally. The articular and periarticular erosion with joint soft tissue thickening pointed to an inflammatory joint arthropathy. Gout was considered instead of pseudogout because of the lack of soft tissue calcifications, pseudogout tends to cause soft tissue calcifications. A CT-guided biopsy of the soft tissue thickening and aspiration of the joint synovial fluid was performed.

Sodium urate crystals were detected under polarised light microscopy. Histopathology showed mixed acute and chronic inflammation with amorphous basophilic and eosinophilic material. No malignancy was detected. Diagnosis of gout of the left TMJ was made based on microscopy findings.

## Treatment

The patient was commenced on a tapering prednisolone regime and planned for urate lowering therapy with allopurinol after 2 weeks and clinical follow-up in 3 months. A total joint replacement was considered concurrently by the treating TMJ surgeon due to the degree of TMJ destruction and skull base erosion. However, the patient had significant comorbidities and surgery was regarded as high risk. Hence, surgery was reserved for persistence or progression of symptoms despite the initial medical therapy as the patient had preserved joint function. The decision on surgery at follow-up will be based on a risk–benefit analysis after re-evaluating the patient. Unfortunately, the patient passed away from an unrelated cardiac event 2 weeks after the diagnosis of gout had been made.

## Discussion

The incidence of gout increases with age and is more common in males and post-menopausal female.^5^ The four phases of gout are asymptomatic hyperuricaemia, acute joint arthritis, intercritical gout and chronic tophaceous gout.^5^ The classic presentation of gout is an acute monoarthritis affecting the first metatarsophalangeal joint but it can also commonly involve the upper extremities and can progress to be polyarticular. The differential diagnosis of isolated TMJ pain include myofascial pain, internal derangement, trauma, osteoarthritis, rheumatoid arthritis and, less commonly, neoplasms and pseudotumours such as synovial chondromatosis.^4^ It is unusual for gout to present in the TMJ, with only 14 cases reported in English literature and 3 involving the skull base.^1,4,8^

The approach to diagnosing gout of the TMJ varies in the literature. Oliveira et al argue that diagnosis can be made with a previous history of gout, presence of hyperuricaemia and imaging, without the need for a biopsy.^1^ In our case, the patient had no previous history of gout and conventional imaging was insufficient to confirm a diagnosis. Malignancy was considered at first. The initial CT and MRI (performed and reported externally) reports did not include a suspicion for gout and it was only considered after an experienced head and neck radiologist reviewed the images. Conventional CT and MRI are non-invasive techniques that can assess the degree of delineation, arthrosis, erosion and soft-tissue changes, but are not specific in diagnosis unless nodules are calcified and may be mistaken for other diseases like malignancy or pseudogout.^2–4,8^

**Figure 3.**
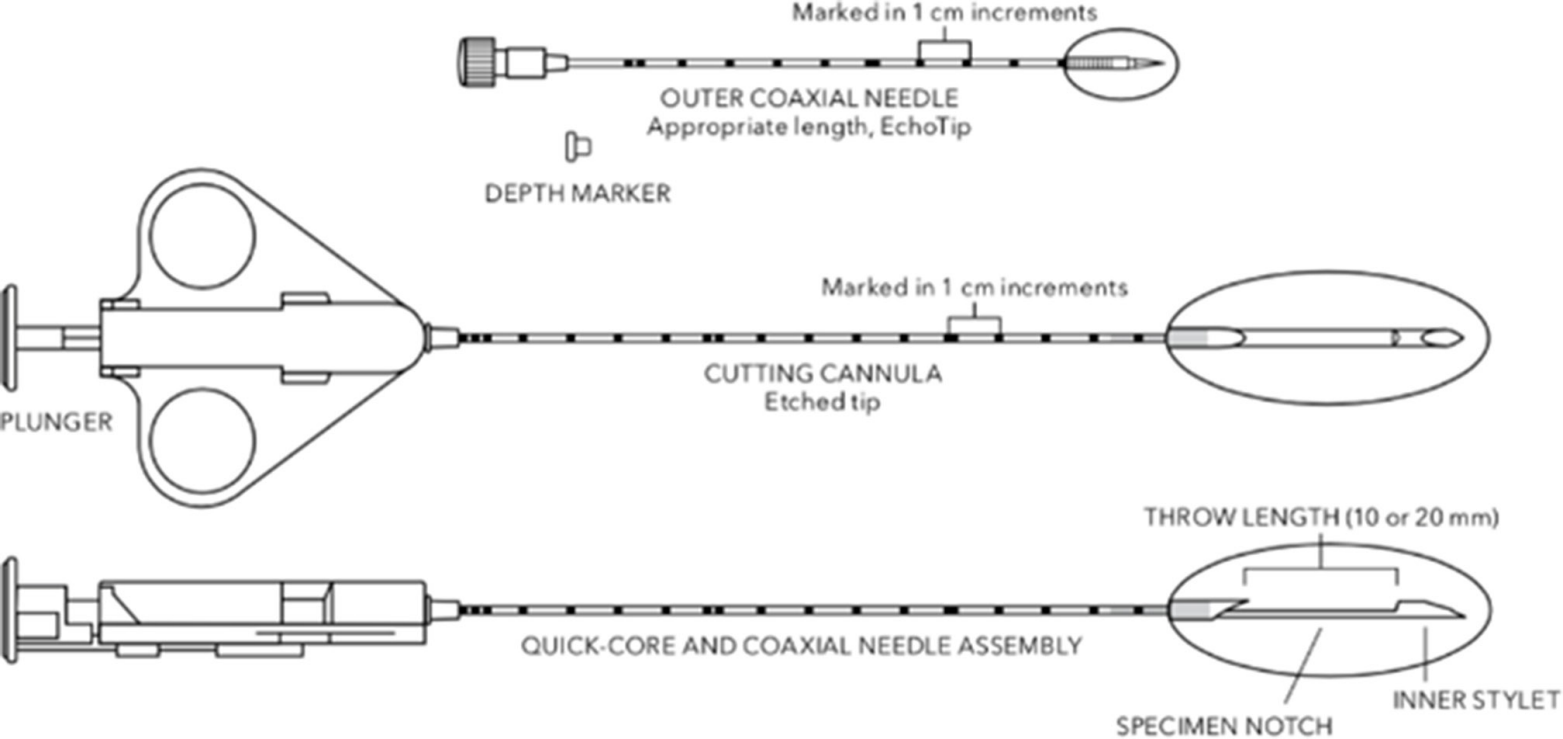
Quick-Core Biopsy Needle set, containing a Quick-Core needle and a coaxial outer needle. The specimen notch can be opened by retracting the cutting cannula to 10 mm or 20 mm in length, depending on the size of the lesion.^10^

It is well-known that the gold-standard for diagnosis of gout is arthrocentesis of the affected joint to visualise MSU crystals.^5^ Some factors affect the feasibility to biopsy, including the general health of the patient and location of the affected joint. Our patient was unsuitable for an open surgical biopsy due to her comorbidities and an unguided biopsy in an outpatient setting would be unsafe due to the deep location of the lesion and potentially nondiagnostic if poorly sampled. Hence, we opted for a CT-guided biopsy of the TMJ. Theoretically, when performing a biopsy of the TMJ, structures at risk of injury include the joint capsule, articular disc and ligaments, however in our patient’s situation, they were already damaged by the pathology and therefore, the benefit of the biopsy far outweighed the potential risk. Generally, inserting the biopsy needle to reach the target lesion carries minimal risk, as the needle tip tends to displace mobile structures like nerves, rather than cutting them. Strategies to optimise pathway to lesion include patient positioning, needle type and performing multimodality imaging.^9^ In our case, the patient was positioned in a supine position with the head titled to the opposite side to maximise comfort. A 14-gauge Quick-Core Biopsy Needle was passed through a 13-gauge coaxial guide into the soft tissue thickening. The radiologist ensured the needle tip did not extend beyond the target lesion. This reduced the risk of complications, as when taking biopsies, the specimen notch remains stationary and only the cutting canula moves, closing over the specimen notch and enclosing the sample as it does so (Figure 3). A pitfall of CT-guided biopsy is the lack of real-time imaging leading to inaccuracies. Adjunct or multimodality imaging such as using ultrasound can help localise the lesion and facilitate needle insertion if CT alone is inadequate.^9^ CT-guided biopsies have not been previously reported in English literature in detecting gout of the TMJ. Not all hospitals and radiology departments are comfortable or experienced with performing such biopsies, and we recommend referral of such cases to centres with experienced head and neck radiologists as nondiagnostic biopsies can complicate or delay appropriate care.

The therapeutic goals for gout of the TMJ are the same as for gout affecting other joints: pain management with colchicine, steroids and NSAIDs, lowering serum urate levels with allopurinol and regular monitoring for disease progression.^4,5,7^ The severity of pain, degree of TMJ dysfunction and any resultant dentofacial deformity will dictate the need for surgical intervention.^7^ Surgery was performed in 8 of the 14 cases of gout of the TMJ, and 2 of the 3 cases involving the skull base, with favourable outcomes in improvement of joint function and correction of deformity.^2–4,8^ Surgery ranged from arthroplasty to partial or total joint replacement with either autogenous grafts or alloplastic material.^7^ Given the degree of TMJ destruction of the patient, if she had a lower risk of intraoperative complications, we would have recommended a total joint replacement to: (i) seal the skull base to prevent neurological complications and provide a stable fossa, (ii) replace the condylar head to regain any lost height. Due to the small sample size of gout of the TMJ especially with skull base involvement, we encourage clinicians to report their cases in the literature to build an evidence base about this disease presentation.

In conclusion, this case demonstrates the importance of considering gout in TMJ disease, particularly when image findings demonstrate erosive and destructive changes progressing to the skull base. These cases may be best managed in a centre with experienced head and neck radiologists and TMJ surgeons. CT-guided biopsy, in experienced hands, is quick, minimally invasive and facilitates diagnosis and treatment planning. Surgery should be considered in cases with skull base involvement, severe deformity, and if pain or joint dysfunction persist despite medical management.

## Learning points

- Gout of the TMJ should be considered in patients presenting with isolated TMJ pain to avoid suboptimal management. It can cause erosion into the skull base, although uncommon.
- CT-guided biopsy of the TMJ by an experienced head and neck radiologist can be performed instead of a more invasive and time-consuming open surgical biopsy to facilitate diagnosis.
- Surgery can be considered in patients presenting with skull base involvement, severe deformity, or have failed medical therapy to prevent neurological complications, treat TMJ dysfunction and manage pain.
